# Poster Session I - A73 OVERCOMING PHYSICAL BARRIERS TO ENDOSCOPY WITH AN ADAPTIVE SUPPORT DEVICE FOLLOWING A LEFT UPPER EXTREMITY INJURY

**DOI:** 10.1093/jcag/gwaf042.073

**Published:** 2026-02-13

**Authors:** G S Sambhi, W Manishen, A Ilnyckyj

**Affiliations:** Gastroenterology, University of Manitoba Max Rady College of Medicine, Winnipeg, MB, Canada; Gastroenterology, University of Manitoba Max Rady College of Medicine, Winnipeg, MB, Canada; Gastroenterology, University of Manitoba Max Rady College of Medicine, Winnipeg, MB, Canada

## Abstract

**Background:**

Endoscopy is a physically demanding procedure that requires normal range of motion and strength of the upper limbs. An upper extremity injury can significantly impair an endoscopist’s ability to perform procedures, necessitating interruption of their endoscopic practice. We present a case of a novel application of an ENT scope holder being used to enable an endoscopist recovering from a fractured left elbow to continue performing procedures safely and effectively. A gastroenterologist at our institution sustained a fractured left proximal radius and ulna requiring operative fixation with replacement of the radial head with a rod and pin/plate stabilization of the olecranon. During the rehabilitation phase of the injury, at week 10 post-surgery, they could hold and manipulate a gastroscope. However, fatigue and discomfort of the injured extremity made consecutive or longer cases challenging.

**Aims:**

To explore adaptative technologies that can be used to assist endoscopists with upper extremity injuries.

**Methods:**

We rested the head of the Olympus GIF-HQ190 gastroscope in the Olympus MAJ-2145 ENT scope hanger. By placing the scope in the scope hanger for the duration of the procedure, the endoscopist was able to reduce left upper extremity fatigue and discomfort. We repositioned the patient’s bed and endoscopy tower to accommodate this setup.

**Results:**

With the endoscope suspended, the endoscopist could use their left hand to effectively control the wheels and buttons while resting the left arm on the cart table. The right hand was able to apply torque to the scope. Although, this device allowed the endoscopist with an injured left hand to continue scoping, there were some challenges. Notably, the length of the scope posed an issue. Since the endoscope was placed farther from the patient’s mouth than normal, we began running out of instrument shaft by the time we reached the pylorus, even without looping. We were able to compensate for this by ensuring the patient’s bed and mouth were very close to the tower.

**Conclusions:**

This case illustrates a novel use of the Olympus MAJ-2145 ENT scope hanger to enable an endoscopist with a left upper extremity injury to continue performing gastroscopies while limiting fatigue and pain during the procedure. We found that this device was most effective for diagnostic procedures where procedure time is short. This case highlights the potential role of an existing device being adapted to support endoscopists with functional limitations. Although in this case the endoscopist had a fractured elbow, we suspect benefit might be found for those with a frozen shoulder or with lateral epicondylitis.

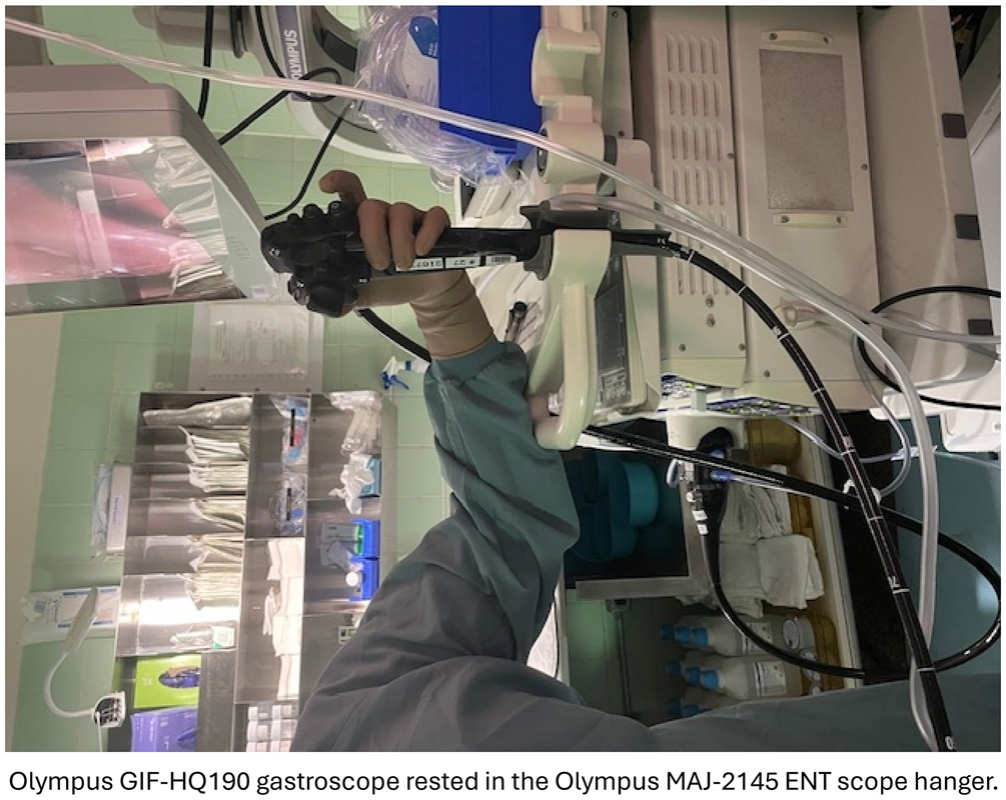

**Funding Agencies:**

None

